# Evaluation of Serum 25(OH) Vitamin D as a Risk Factor in Adult Recurrent Tonsillitis

**DOI:** 10.7759/cureus.32083

**Published:** 2022-11-30

**Authors:** Hossam A Hussein, Ali M Alqannass, Mohammed H Al Mansour, Ahmed A Safhi

**Affiliations:** 1 Otorhinolaryngology, King Khalid Hospital, Najran, SAU; 2 Otorhinolaryngology, Menoufiya University, Shebien Elkoom, EGY; 3 Otorhinolaryngology, Najran General Hospital, Najran, SAU

**Keywords:** vitamin d deficiency, recurrent tonsillitis, risk factor, adults, 25-hydroxy vitamin d

## Abstract

Background

Studies have reported that the rate of upper respiratory tract infections in children and adults is inversely related to serum vitamin D levels and supplementation with vitamin D reduces the incidence of this infection. This study aimed to examine if vitamin D serum levels were a risk factor for recurrent tonsillitis among adult patients.

Methods

Retrospective comparative analysis was carried out on 100 patients diagnosed with recurrent tonsillitis with 100 age- and sex-matched individuals as controls between June 2016 and May 2022. Tonsillar size was assessed based on Brodsky grading system. Serum levels of 25-hydroxy (25(OH)) vitamin D, total calcium, iron, hemoglobin, C-reactive protein (CRP), and erythrocyte sedimentation rate (ESR) were analyzed.

Results

There was a statistically significant low vitamin D levels in tonsil size grades III+IV compared to grade I+II (p <0.001) among cases. There was a significantly lower serum value for 25(OH) vitamin D in the cases as compared to the control group (p <0.001). A high percentage (68%) of cases had vitamin D deficiency (<20 ng/mL) and this was statistically significant (p <0.001). There were statistically significant higher values of CRP and ESR among cases compared to the control group (p <0.001). At a cut off (≤21.2), serum vitamin D levels achieved 78% sensitivity, 65% specificity (p <0.001) to differentiate cases from controls. Following logistic regression analysis, the level of vitamin D was the only significant risk factor.

Conclusion

Findings from our study also suggest an association between recurrent tonsillitis among our adult cohorts and low serum 25(OH) vitamin D levels. Therefore, we opine that serum vitamin D levels should be considered in the management of adult patients with tonsillitis.

## Introduction

In the upper respiratory tract (URT) is a ring of specialized lymphoid organs (also known as Waldeyer's ring) that houses the palatine tonsils, one of its major components. Infections and hypertrophy of the palatine tonsils make up the diseases most commonly seen in otolaryngology practice [1].

The term “recurrent tonsillitis” means recurrent throat infection with a frequency of at least seven tonsillitis bouts in one year, at least five bouts in each of the preceding two years, or at least three bouts in each of the preceding three years with recording (as an option) in the medical file for each bout of sore throat with one or more of the following: fever >38.3°C, cervical lymphadenopathy, tonsillar exudate, or positive group A beta-hemolytic streptococcal test [2,3].

Viral infection accounts for 50-80% of cases, but around 5-17% of cases are bacterial, with group A beta-hemolytic streptococci (GAS) responsible for the majority of cases [3,4]. In adults, recurrent tonsillopharyngitis is considered a major health issue as well as a financial burden due to its frequency, resulting in job loss and leading to unreasonable antibiotic usage [5]. The precise cause of this phenomenon remains unknown, even though that several factors exist to explain the frequent tonsillitis episodes. These include patient non-compliance, premature cessation of anti-biotherapy, insufficient absorption of antibiotics, bacterial tolerance, burden, and biofilms, and immune system dysfunction [4,6].

Furthermore, studies have linked the condition to sunlight deprivation during the winter, which impairs the body's ability to synthesize vitamin D during that particular season [3]. Within the immune system vitamin D plays an essential role, it affects immunological cells, such as B and T lymphocytes, in a variety of ways. It is thought to have immunomodulatory effects on adaptive and innate immunity since it has a role in converting T helper cells into T helper 2 status [3]. Moreover, vitamin D plays an important role in inducing antimicrobial peptides (AMPs), β-defensin and cathelicidin, when the immune system recognizes invading viral or bacterial organisms [6,7]. These peptides represent the two primary defensive agents of URT [8]. In tissues, these peptides directly inhibit the growth of microorganisms exhibiting a broad-spectrum antimicrobial activity [6,7,9]. Oil-soluble vitamin D is produced in the skin in response to sunshine exposure and is found in the diet and is also crucial for the skeletal system's mineralization of bones [10].

Similarly, epidemiological studies have shown that rate of URT infections in children and adults is inversely related to vitamin D levels [7,11,12]. More so, randomized controlled studies have also reported that vitamin D supplementation diminishes the incidence of URT infections [13,14], while other studies showed no significant difference [15,16].

Despite this plethora of research in the literature discussing the possible association between vitamin D and recurrent tonsillitis, only a handful of studies had been undertaken in adults. To our knowledge, this paper represents the first study in the Kingdom of Saudi Arabia (KSA) aiming to evaluate vitamin D as a risk factor for recurrent tonsillitis in adults.

## Materials and methods

Eligibility criteria for participants

One hundred adult patients with recurrent tonsillitis were recruited as cases (group 1), while the control (group 2) consisted of 100 subjects with no prior history of recurrent tonsillitis randomly selected from the Otorhinolaryngology (ORL) outpatient clinic of King Khalid Hospital (KKH), Najran-KSA, between June 2016 and May 2022. Cases and controls were selected by simple random sampling via computer-generated random numbers after assigning numbers to attendees throughout study period.

Using current clinical guidelines eligible cases were recruited [2]: seven or more tonsillitis bouts in one year or five or more tonsillitis bouts in each of the preceding two years or three or more tonsillitis bouts in each of the preceding three years. Patients with allergic rhinosinusitis, systemic disorders, chronic conditions other than chronic or recurrent tonsillitis, or age <18 years were excluded. Similarly, patients who had recently taken vitamin D supplements or consumed low-calorie diets in the past year, as well as those with uncontrolled thyroid or parathyroid conditions or any patient with incomplete and/or missing data (five patients had incomplete data and 11 had missing data).

While for the control group, the following conditions were the exclusion criteria: tonsillectomy or a history of frequent tonsillitis; any known chronic systemic disorder; taking vitamin D supplements, and a history of frequent URT infections. There were no missing/incomplete data for the control group.

Study design

Gender, age, total calcium, serum iron, hemoglobin, C reactive protein (CRP), erythrocyte sedimentation rate (ESR), and serum levels of 25-hydroxy (25(OH)) vitamin D were compared between cases and controls in a retrospective study.

From each participant’s medical records (Oasis medical records system) in both groups, details on clinical evaluation including Brodsky tonsil size grading, illnesses, pharmacological treatments, and the outcomes of laboratory testing were retrieved.

Laboratory procedures

Each participant provided 5 milliliters sample of venous blood under complete aseptic conditions. A Sysmex XN-3000 (Kobe, Japan) was used for complete blood count (CBC) measurement by injecting 1 mL of blood into an EDTA-containing tube. The Westergren method was applied for ESR measurement by injecting 1.6 mL of blood along with 0.4 mL of sodium citrate into a tube [17]. 2 mL of blood was delivered in a plain tube, left for 30 minutes to clot, and then centrifuged at 4000 rpm for 10 minutes; the yielded serum was used for CRP measurement using enzyme-linked immunosorbent assay (ELISA). Serum iron and calcium were measured by DxC 700 AU (Beckman Coulter, Brea, CA, USA). Quantitative determination of the total 25(OH) vitamin D level in human serum was done using the DxI Immunoassay Systems (Access 25(OH) vitamin D Total, Unicel DxI 800, Beckman Coulter). Values falling within the following ranges CRP 0-0.9 mg/L, ESR 0-20 mm/hr., male hemoglobin 12.1 to 15.5 g/dL, female hemoglobin 13.6 to 15.1 g/dL, serum iron 8.1-32.6 umol/L, and serum total calcium 2.2-2.58 mmol/L were all regarded normal values for laboratory testing. According to Endocrine Society guidelines, 30-100 ng/mL is the accepted range for serum 25(OH) vitamin D levels while insufficiency is recognized as levels below 30 ng/mL and deficiency as levels below 20 ng/mL [18].

Statistical analysis of the data

With the aid of the SPSS statistical software package version 20.0 (IBM Corp, Armonk, NY, USA), data were fed into the computer and evaluated. Frequencies and percentages were measured to describe categorical variables. In order to examine the relationship between the categorical variables, the chi-square test was employed. If more than 20% of the cells have an expected count of less than five, the Monte Carlo correction test was instead used. The Kolmogorov-Smirnov test was applied to test the normality of continuous data, and the range (minimum and maximum), mean, standard deviation, and median were used to express the quantitative data. The diagnostic performance of the markers was evaluated using the receiver operating characteristic curve (ROC); an area greater than 50% indicates an acceptable level of performance, while an area around 100% indicates the test's optimal performance. Level of statistical significance was judged at p ≤ 0.05.

Ethical considerations

According to International Conference on Harmonisation - Good Clinical Practice (ICH GCP) guidelines, the research protocol was reviewed and approved by the Institutional Review Board (IRB) at the Directorate of Health Affairs in Najran under IRB Log Number 2022-58 E and IRB registration number with KACST- KSA: H-11-N-081.

## Results

In this study, there was no significant difference between both study groups regarding age and gender (p = 0.179 and 0.089 respectively) (Table 1). With regard to tonsil size based on Brodsky grading, majority of the cases had grade II (44%) (Table 1).

**Table 1:** Comparison between the two studied groups according to demographic and laboratory data. IQR: Inter quartile range, CRP: C-reactive protein, ESR: erythrocyte sedimentation rate, SD: Standard deviation, X^2^: Chi-square test, U: Mann-Whitney U test, t: Student t-test, p: p value for comparing between the studied groups *: Statistically significant at p ≤ 0.05

	Cases (n = 100)	Controls (n = 100)	Test of Sig.	p
Gender				
Male	47(47%)	59(59%)	X^2^= 2.890	0.089
Female	53(53%)	41(41%)		
Age (years)				
Median (IQR)	27 (22 – 31.5)	29.5 (24 – 34)	U= 4451.0	0.179
Brodsky Grading (Tonsil Size)				
I	14(14%)	–	–	–
II	44(44%)	–		
III	39(39%)	–		
IV	3(3%)	–		
Serum 25(OH) Vitamin D (ng/mL)				
Median (IQR)	16 (12 – 21)	24.8 (16.2 – 29.1)	U= 2784.0^*^	<0.001^*^
Serum 25(OH) Vitamin D Level				
Deficiency (< 20 ng/mL)	68(68%)	32(32%)	X^2^= 25.941^*^	<0.001^*^
Insufficiency (20-30 ng/mL)	24(24%)	50(50%)		
Normal (>30-100 ng/mL)	8(8%)	18(18%)		
Total Calcium (mmol/L)				
Mean ± SD.	2.3 ± 0.1	2.5 ± 0.6	t= 1.902	0.060
Serum Iron (umol/L)				
Median (IQR)	9 (5.8 – 12.7)	11 (6.4 – 15)	U= 4388.0	0.134
CRP (mg/L)				
Negative	31(31%)	87(87%)	X^2^= 64.820^*^	<0.001^*^
Positive	69(69%)	13(13%)		
ESR (mm/hr)				
Median (IQR)	18.5 (10 – 35.5)	7 (4 – 12)	U= 2192.0^*^	<0.001^*^
Hemoglobin (g/dL)				
Mean ± SD.	13.5 ± 2	13.9 ± 1.9	t= 1.635	0.104

This study showed significantly lower serum 25(OH) vitamin D values in the cases compared with its levels in the control group (p <0.001). In terms of serum 25(OH) vitamin D level, the distribution of cases and controls varied significantly (P <0.001) in which, majority of the cases had vitamin D deficiency (<20 ng/mL) (68%) (Table 1).

Our results showed significantly higher values of CRP and ESR in the patient group compared with the control group (p <0.001) although there was no significant difference between the cases and the control group regarding total calcium, serum iron, and hemoglobin (p = 0.6, 0.134, and 0.104, respectively) (Table 1).

There was a significant relationship between serum 25(OH) vitamin D and tonsil size (Brodsky grading system) with lower levels of vitamin D in patients with tonsil size grade III+IV compared to grade I+II (p <0.001). All patients with grade III+IV (100%) documented vitamin D deficiency, while the percentages of patients with grade I+II and vitamin D deficiency, vitamin D insufficiency, and normal vitamin D were 44.8%, 41.4%, and 13.8%, respectively (p <0.001) (Table 2).

**Table 2:** Relation between Brodsky grading (tonsil size) and serum 25(OH) vitamin D in cases group. X^2^: Chi-square test, MC: Monte Carlo, U: Mann-Whitney U test, p: p value for comparing between the studied groups *: Statistically significant at p ≤ 0.05

	Brodsky Grading (Tonsil Size)		Test of Sig.	p
	I+ II (n= 58)	III + IV(n= 42)		
Serum 25(OH) Vitamin D (ng/mL)				
Median (Min. – Max.)	20(9 – 57)	12(9 – 19)	U= 244.50^*^	<0.001^*^
Serum 25(OH) Vitamin D Level				
Deficiency (< 20 ng/mL)	26(44.8%)	42(100%)	X= 39.454^*^	^MC^p <0.001^*^
Insufficiency (20-30 ng/mL)	24(41.4%)	0(0%)		
Normal (>30-100 ng/mL)	8(13.8%)	0(0%)		

**Figure 1:**
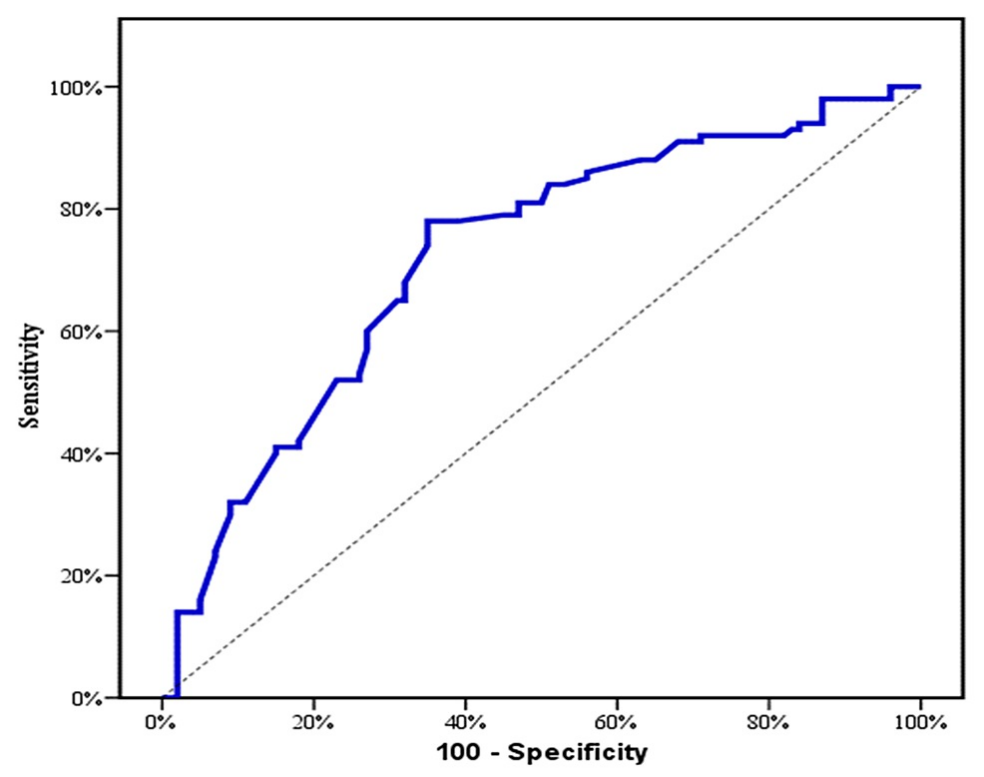
Receiver operating characteristic (ROC) curve for serum 25(OH) vitamin D to discriminate cases from controls.

**Figure 2:**
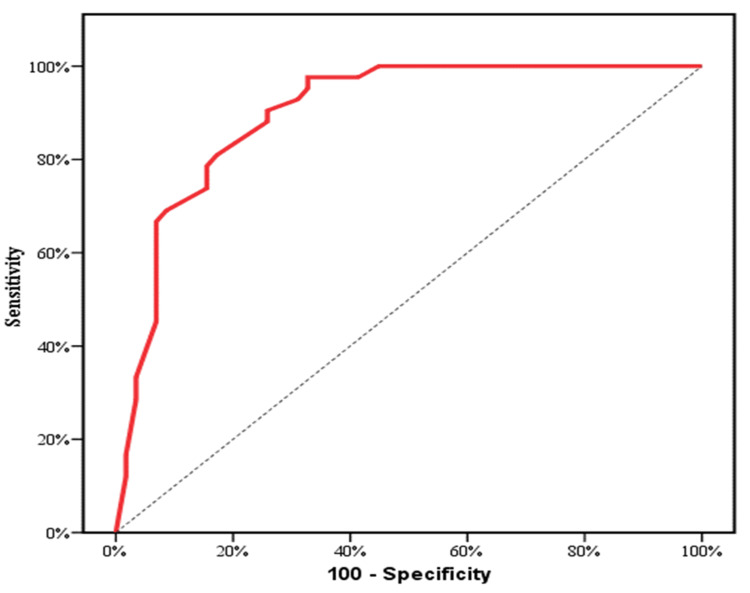
Receiver operating characteristic (ROC) curve for serum 25(OH) vitamin D to discriminate III +IV from I + II.

The prognostic power for serum 25(OH) vitamin D was evaluated by ROC curve analysis. At a cut off (≤ 21.2), serum 25(OH) vitamin D achieved 78% sensitivity, 65% specificity, area under the curve (AUC) of 0.722 and (p <0.001) to discriminate cases from controls (Table 3, Figure 1). At a cut off (≤ 16.2), serum 25(OH) vitamin D displayed 90.48% sensitivity, 74.14 specificity, AUC of 0.900 and (p <0.001) to discriminate patients with grade (III +IV) from those with grade (I + II) (Table 3, Figure 2).

**Table 3:** The prognostic performance for serum 25(OH) vitamin D. AUC: Area Under a Curve, p value: Probability value, CI: Confidence Intervals, NPV: Negative predictive value, PPV: Positive predictive value *: Statistically significant at p ≤ 0.05

	AUC	p	95% C.I	Cut off	Sensitivity	Specificity	PPV	NPV
to discriminate cases (n= 100) from controls (n= 100)								
Serum 25(OH) Vitamin D (ng/mL)	0.722	<0.001^*^	0.651 – 0.792	≤21.2	78.0	65.0	69.0	74.7
to discriminate III +IV (n= 42) from I + II (n= 58)								
Serum 25(OH) Vitamin D (ng/mL)	0.900	<0.001^*^	0.840 – 0.960	≤16.2	90.48	74.14	71.7	91.5

Using logistic regression analysis for the parameters affecting recurrent tonsillitis in adults, it appeared that the level of serum 25(OH) vitamin D was the only statistically significant risk factor (Table 4).

**Table 4:** Logistic regression analysis for the parameters affecting recurrent tonsillitis in adults. OR: Odds ratio, CI: Confidence interval, LL: Lower limit, UL: Upper Limit #: All variables with p<0.05 were included in the final multivariate regression model *: Statistically significant at p ≤ 0.05

	p	OR (LL – UL 95%C.I)
Gender	0.609	1.231(0.555 – 2.728)
Age (years)	0.086	0.950(0.897 – 1.0)
Serum 25(OH) Vitamin D	<0.001^*^	0.645(0.541 – 0.768)
Total Calcium (mmol/L)	0.313	4.882(0.225 – 105.940)
Serum Iron (umol/L)	0.607	0.978(0.899 – 1.064)
CRP (mg/L)	0.993	1.004(0.425 – 2.369)
ESR (mm/hr)	0.468	1.010(0.984 – 1.036)
Hemoglobin	0.573	1.059(0.869 – 1.290)

## Discussion

Within the scope of our literature knowledge, few studies have investigated the relationship between vitamin D levels and recurrent tonsillitis among adult patients worldwide. To our knowledge, this is the first study reported in KSA evaluating vitamin D as a risk factor for recurrent tonsillitis in adults.

Serum levels of 25(OH) vitamin D were preferentially measured due to its 20-day serum half-life, which is longer than that of the active form of vitamin D, 1,25(OH)2, which has a half-life of only three to six hours. This has been quoted as a standard reference for the assessment of vitamin D levels by several researchers [1,19].

Our study and that by Al-Rawashdeh et al. [8] showed no statistically significant difference between both study groups regarding age and gender. Whereas, Elbistanlı et al. and Science et al. both reported that young age and the reduction of vitamin D levels were linked to a higher rate of URT infections [6,20].

We documented the median serum 25(OH) vitamin D level in the recurrent tonsillitis group as significantly lower than that of the control group (16 ng/mL and 24.8 ng/mL respectively). Additionally, more than two-thirds (68%) of the recurrent tonsillitis group had vitamin D deficiency compared to nearly one-third (32%) of the control group. These findings suggest that recurrent tonsillitis bouts were inversely proportional to vitamin D levels, and these were also in line with the findings of Al-Rawashdeh et al. [8] and Safak et al. [21], which showed a significant relationship between low vitamin D levels and recurrent tonsillitis. In another study, low serum vitamin D levels were linked to an increased risk for URT infections, using polymerase chain reaction (PCR) assay. Serum vitamin D levels below 28 ng/mL were found to increase the risk of URT infections by (50%), whereas levels below 20 ng/mL increased the risk by up to (70%) [20]. In contrast, Aydın et al. in their study reported no significant difference between the recurrent tonsillitis group and the control group in regard to mean vitamin D serum levels. Although, patients with recurrent tonsillitis had a higher rate of vitamin D insufficiency [22]. This difference may not be unrelated to age of participants (among pediatric age groups) and utilization of PCR as well as vitamin D receptor gene polymorphisms which may probably confer better fidelity in their analysis.

Regarding inflammatory markers, CRP and ESR, our study revealed significantly higher values in adults with recurrent tonsillitis compared with the control group. These markers are usually elevated in patients with recurrent tonsillitis and are of help in monitoring their surgical treatment [23]. Moreover, CRP has an additional clinical utility in distinguishing the microbial etiology by being significantly higher in streptococcal tonsillitis than other throat infections [24,25].

Our study also showed that trace elements (such as iron) were not associated with recurrent tonsillitis as serum iron and hemoglobin levels were normal. Iron deficiency has been linked to abnormal cell-mediated immune responses and a reduced ability of phagocytic cells to eradicate particular bacterial species [26]. Although our findings do not align with previous studies in which low iron levels have been linked to recurrent tonsillitis [27,28]. Elverland et al. reported that iron and hemoglobin metabolism were improved after tonsillectomy and adenoidectomy, and observed that patients with recurrent tonsillitis and upper airway obstruction usually had low serum iron levels [29].

Concerning tonsillar size, we reported a significant relationship between serum vitamin D levels and tonsil size where lower levels of vitamin D were observed in patients with tonsil sizes of grade III+IV compared to grade I+II. Other studies have reported that vitamin D levels were negatively associated with the sizes of tonsils [6,30]. This may be explained using a mouse model [31], that vitamin D receptor-deficient mice exhibit an accumulation of mature dendritic cells, leading to subcutaneous lymph nodes enlargement. Furthermore, low vitamin D levels may contribute to adenotonsillar enlargement by reducing the suppression of dendritic cell maturation [32]. However, another study concluded that there was no correlation between vitamin D status and tonsil size [1].

Using logistic regression analysis for the parameters affecting recurrent tonsillitis in adults, it was observed that the level of serum 25(OH) vitamin D was the only significant risk factor. Although there has long been concern that a lack of vitamin D increases the risk of respiratory tract infections, to the best of our knowledge this relationship has not been proven separately and deeply among adult patients, particularly in KSA.

This study has a few potential limitations. Only one serum vitamin D measurement per subject was obtained, not taking into cognizance the seasonal changes in its levels. Ideally, this should be measured during the cold season as well. Moreover, the sample size of our study may be too small to make population-based inferences. Similarly, there are other factors associated with vitamin D levels that were not considered such as dietary habits, sleeping habits, body fat, time spent outside, overall fitness, etc., which may predispose to some bias.

In the future, we hope to evaluate whether providing supplementation could reduce the recurrence of tonsillitis among adult patients.

Therefore, our finding suggests an association but it may be premature to extend this finding to the general population. Hence, there is a need for further research to be conducted in one season with larger sample sizes of adult patients and multicentric levels to strengthen the hypothesis regarding the association between serum vitamin D levels and ‘adult’ recurrent tonsillitis.

## Conclusions

Our study lays credence to a possible relationship between adult patients with recurrent tonsillitis and low vitamin D levels and this may also be linked to variable tonsillar sizes. As a result, we propose that testing of serum 25(OH) vitamin D level in adults with recurrent tonsillitis should be considered as part of the management strategy.
